# Sarcomatoid lung carcinomas: a case series

**DOI:** 10.4076/1757-1626-2-7900

**Published:** 2009-06-11

**Authors:** Panagiotis Hountis, Sotirios Moraitis, Panagiotis Dedeilias, Polychronis Ikonomidis, Mattheos Douzinas

**Affiliations:** Department of Thoracic and Cardiovascular Surgery, Athens Naval and Veterans HospitalDeinokratous 70 Kolonaki, AthensGreece

## Abstract

We report on three Caucasian Greeks 2 males and 1 female (67, 54 and 62 years old) that were operated with sarcomatoid carcinoma of the lung, an uncommon tumor that sometimes is referred as pleomorphic carcinoma (spindle and giant cell carcinomas). These tumors are encountered in the thorax far more often than true sarcomas. There are many erroneous reports of pulmonary sarcomas made before the advent of adjunctive pathologic screening, including immunohistochemical studies. Pulmonary Sarcomatoid Carcinomas represent 0.2-1% of all lung cancers in different series and they are considered that they are not significantly aggressive than ordinary lung carcinoma.

## Introduction

Sarcomatoid carcinomas (SC) of the lung (spindle and giant cell carcinomas) are rare histologic subtypes and have been reported as having a poor prognosis. In the World Health Organization (WHO) histologic classification of lung tumors, spindle cell carcinoma is classified as a variant of squamous cell carcinoma and giant cell carcinoma is classified as a variant of large cell carcinoma. Fishback et al. designated sarcomatoid carcinomas as pleomorphic (spindle/giant cell) carcinomas of the lung because both spindle and giant cell components are often found in the same tumors [1].

## Case presentation

Since 1990 three Caucasian Greeks 2 male and 1 female (67, 54 and 62 years old) were operated at the Department of Thoracic and Cardiovascular Surgery at Athens Naval and Veterans Hospital with a final diagnosis of SC. All had a history of cigarette smoking (60, 45, 25 pack/years respectively) and social alcohol drinking but without significant medical or family history. All three had a normal weight for their height. The male patients were former shipyard workers and the female an office worker. Presenting symptoms were cough, hemoptysis and atypical chest discomfort. In one case, the presenting symptom was diarrhea. Two of the tumors were in the right upper lobe and one case in left lower lobe. All of the patients had a complete pre-operative staging with CT scans, bronchoscopy and radioisotopic studies of the body. FNA cytologic examination revealed non-small cell lung cancer.

Right pneumonectomy, right upper lobectomy and left lower lobectomy were performed respectively with mediastinal lymph node dissection, which revealed N2 disease in one case. All 3 patients received postoperative chemotherapy and radiation as a part of an adjuvant therapy in their management.

Immunohistochemical studies were performed by the avidin-biotin-peroxidase complex method. Spindle cell type was comprised of fusiform malignant cells that were positively reactive to one or more epithelial markers including cytokeratins, carcinoembryonic antigen, and epithelial membrane antigen. Giant cell type was comprised of tumor giant cells with abundant cytoplasm and multiple nuclei or a single large pleomorphic nucleus. The mixed type exhibit a mixture of spindle cell type and giant cell type, each of which comprised ≥25% of the sarcomatoid areas. Histology of these specimens was diagnostic for sarcomatoid carcinomas of the lung of giant cell type in one patient and spindle cell type in two, using immunohistochemical studies. One of the patients (male 67 years old) is still alive 21 months after the operation. The other two died 7 months and 12 months after the operation, respectively, from distant metastases to liver and contalateral lung respectively.

## Discussion

Sarcomatoid carcinomas (SC) of the lung are a heterogeneous group of non-small cell lung carcinomas (NSCLC) containing a sarcoma or sarcoma-like component. SC may represent an epithelial neoplasm undergoing divergent tissue differentiation originating from a single clone. These tumors are the most common pulmonary neoplasms that exhibit a composition by spindled or pleomorphic tumor cells. As such, many of them may be confused easily with true sarcomas diagnostically unless special immunohistological or ultrastructural analyses are performed. Accurate diagnostic workup is crucial for the management of these uncommon tumors [2].

Reactivity is expected for keratin, epithelial membrane antigen, or collagen type IV in the sarcoma-like elements in SC, although it may be focal. Electron microscopy often shows the presence of junctional complexes between tumor cells, with or without pericellular basal lamina and cytoplasmic skeins of intermediate filaments. Current terminological preferences are such that several formerly used terms--including “spindle-cell carcinoma,” “pulmonary blastoma,” “squamous cell carcinoma with pseudosarcomatous stroma,” “pseudosarcoma,” and “carcinosarcoma”--are now encompassed by the more generic designation of “sarcomatoid carcinoma.” The clinical course of patients with this neoplasm is aggressive, with an overall 5-year survival rate approximating 20% [3].

With regard to the origin of neoplastic spindle and giant cells, three hypotheses can be considered. First, carcinoma cells change into neoplastic spindle and giant cells and these cells still are carcinoma. This idea is supported by many investigators because the transition of carcinoma cells into spindle cells occasionally is recognized. Second, although these cells originate from carcinoma, they obtain mesenchymal differentiation and change into true sarcoma. Third, these cells originate from sarcoma and differentiate into carcinoma [1].

Although sarcomatoid carcinomas are generally believed to be more aggressive and have a poorer prognosis than ordinary lung carcinomas, no statistical analysis has been reported. Except for the report from the Armed Forces Institute of Pathology by Fishback et al. 2 They reported that tumor size > 5 cm, a clinical Stage > Stage I, and lymph node involvement shortened patient survival, significantly. The median survival of patients with sarcomatoid carcinoma was 10 months, which, when compared with that of ordinary lung carcinomas in the literature (20 months for adenocarcinoma, 18.5 months for squamous cell carcinoma, and 12.6 months for large cell carcinoma), was brief. However, in the current study as in our case series there was no significant difference between the prognosis of patients with sarcomatoid carcinoma and that of patients with nonsarcomatoid, non-small cell carcinoma of the lung [4].

As in every non-small cell lung cancer, early detection, accurate diagnosis and an aggressive operative management is the mainstay of the best chances of long-term survival in these unusual tumors of the lung. The role of adjuvant or neo-adjuvant chemoradiotherapy has not been proved beneficial alone, but they play an important role as an adjunct to standard accepted operative treatment.

## List of abbreviations

CT: Computed Tomography
FNA: Fine needle aspiration
NSCLC: Non-small cell lung cancer
N2: Nodal stations that stage the lung cancer as N2 according to the TNM system
SC: Sarcomatoid Carcinomas
WHO: World Health Organization
